# Crystal structure of 3-(4,4-di­fluoro-5,7-dimethyl-4-bora-3a,4a-di­aza-*s*-indacen-3-yl)propanoic acid

**DOI:** 10.1107/S2056989017016942

**Published:** 2017-11-30

**Authors:** Takuma Kato, Mitsunobu Doi

**Affiliations:** a Osaka University of Pharmaceutical Sciences, Nasahara, Osaka 569-1094, Japan

**Keywords:** crystal structure, fluorescence, BODIPY

## Abstract

The crystal structure of the title compound, C_14_H_15_BF_2_N_2_O_2_, which comprises a boron–dipyrromethene (BODIPY) backbone and a propionic acid group, has been determined at 100 K. The BODIPY fused-ring system is nearly planar, with a maximum deviation from the mean plane of 0.032 (2) Å. In the crystal, pairs of O—H⋯O hydrogen bonds connect the mol­ecules, forming inversion dimers. The dimers are linked *via* C—H⋯O hydrogen bonds, forming a tape along the *a* axis. The tapes are stacked along the *c* axis through C—H⋯F hydrogen bonds and π–π inter­actions.

## Abstract 2

The crystal structure of the title compound has been determined at 100 K. In the crystal, O—H⋯O, C—H⋯On and C—H⋯F hydrogen bonds, and C—H⋯π and π–π inter­actions connect neighbouring mol­ecules.

## Chemical context   

**Figure 1:**
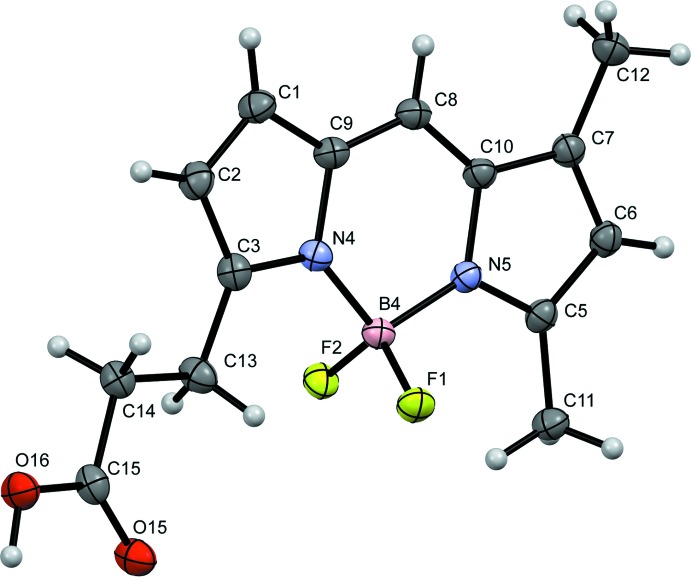
The mol­ecular structure of the title compound, showing the atom-numbering scheme. Displacement ellipsoids are drawn at the 50% probability level and H atoms are shown as small spheres of arbitrary radii.

Boron–dipyrromethene (BODIPY) dyes have promising applications in material sciences for labeling biomolecules such as peptides, proteins, lipids and nucleic acids. BODIPY dyes have many advantages over other dyes, such as robustness against light and chemicals, high absorption coefficients and fluorescence quantum yields, narrow emission bandwidths, and so on (Boens *et al.*, 2012 ▸). Moreover, their spectroscopic and photophysical properties are easy to tune by attachment of some residues at the appropriate positions of the di­fluoro­boron dipyrromethene moiety (Loudet & Burgess, 2007 ▸; Ulrich *et al.*, 2008 ▸). Herein we report the crystal structure of the title compound (Fig. 1 ▸) having the BODIPY fragment.
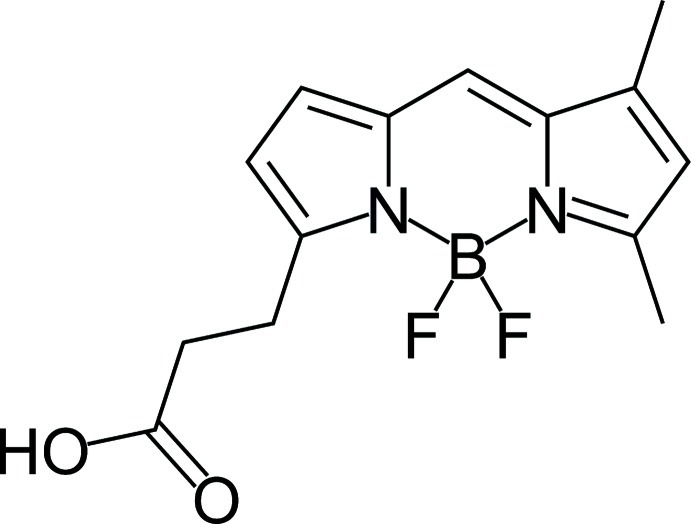


## Structural commentary   

The title compound is composed of a boron–dipyrromethene (BODIPY) backbone and a propionic acid group. The BODIPY fused-ring system is nearly planar, with a maximum deviation from the mean plane of 0.032 (2) Å for atom N4. The bond lengths in the BODIPY framework indicate the strongly delocalized π-system nature [C—C = 1.374 (2)–1.425 (2) Å and C—N = 1.346 (2)–1.401 (2) Å; Fig. 1 ▸]. There are weak intra­molecular C—H⋯F hydrogen bonds present (C11—H11*A*⋯F1 and C13—H13*B*⋯F1; Table 1 ▸).

## Supra­molecular features   

**Figure 2:**
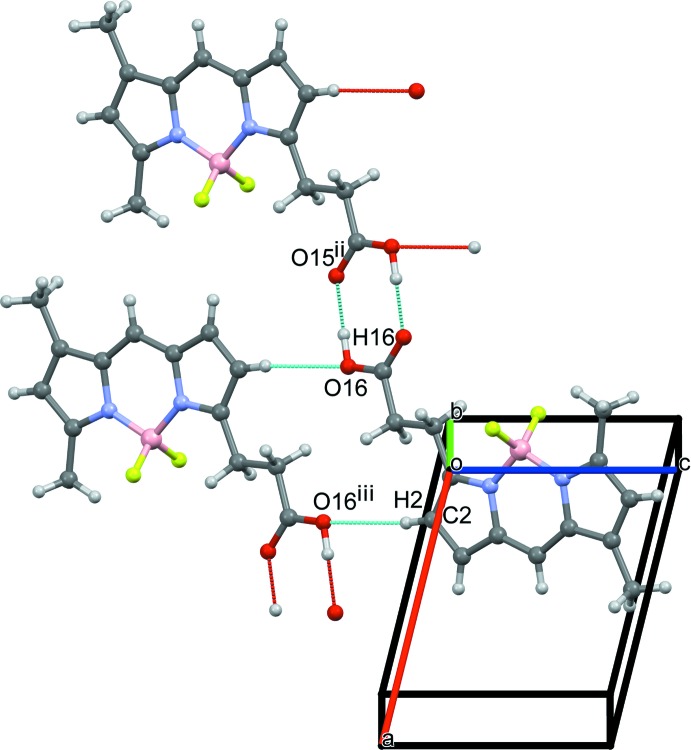
A packing diagram of the title compound, showing the O16—H16⋯O15^ii^ and C2—H2⋯O16^iii^ inter­actions (dashed blue lines). [Symmetry codes: (ii) −*x* + 1, −*y* + 1, −*z* + 3; (iii) −*x*, −*y* + 1, −*z* + 3.]

**Figure 3:**
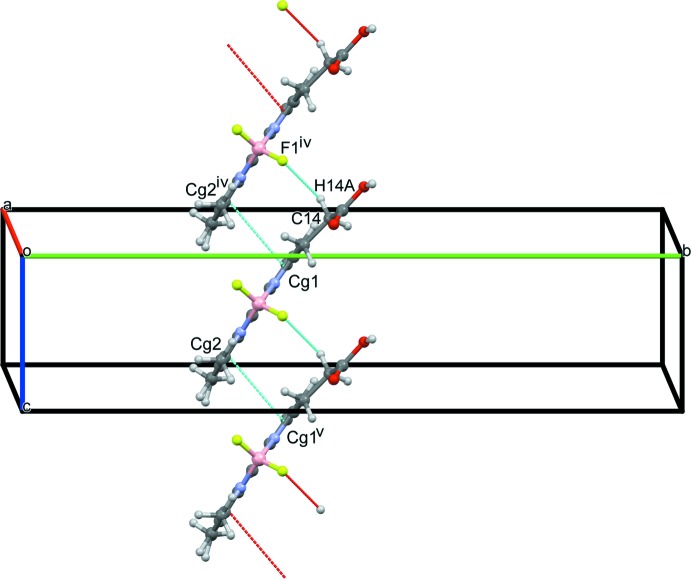
A packing diagram of the title compound, showing the C14—H14*A*⋯F1^iv^ and π–π (*Cg*1⋯*Cg*2^iv^ and *Cg*2⋯*Cg*1^v^) inter­actions (dashed blue lines). [Symmetry codes: (iv) *x*, *y*, *z* + 1; (v) *x*, *y*, *z* − 1.]

**Figure 4:**
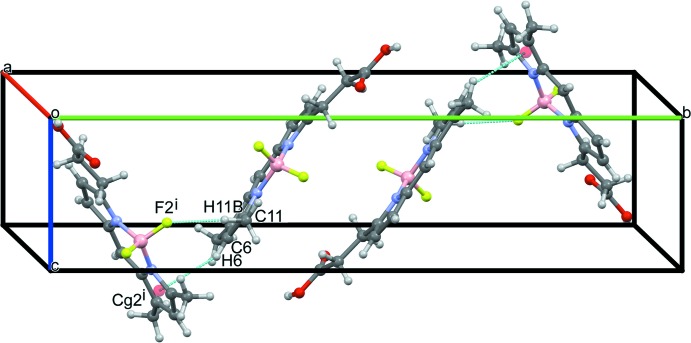
A packing diagram of the title compound, showing the C11—H11*B*⋯F2^i^ and C6—H6⋯*Cg*2^i^ inter­actions (dashed blue lines). [Symmetry code: (i) *x*, −*y* + 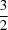, *z* − 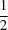.]

Packing diagrams of the title compound are shown in Figs. 2 ▸–4 ▸ ▸. A pair of O—H⋯O hydrogen bonds between the carb­oxy­lic acid groups of opposite-facing mol­ecules connect the two mol­ecules (O16—H16⋯O15^ii^; symmetry code as in Table 1 ▸), forming inversion dimers (Fig. 2 ▸), and these dimers are linked into a tape structure along the *a-*axis direction *via* C—H⋯O hydrogen bonds (C2—H2⋯O16^iii^; symmetry code as in Table 1 ▸). Furthermore, extended stacking of the tapes along the *c-*axis direction forms a layer parallel to the *ac* plane (Fig. 3 ▸) *via* C—H⋯F hydrogen bonds (C14—H14*A*⋯F1^iv^; symmetry code as in Table 1 ▸) and π–π inter­actions [*Cg*1⋯*Cg*2^iv^ = 3.7802 (8) Å; symmetry code: (iv) *x*, *y*, *z* + 1; *Cg*1 and *Cg* 2 are the centroids of the N4/C1–C3/C9 and N5/C5–C7/C10 five-membered rings, respectively]. Between the layers, inter­molecular C—H⋯F and C—H⋯π inter­actions (C11—H11*B*⋯F2^i^ and C6—H6⋯*Cg*2^i^; symmetry code as in Table 1 ▸) are observed (Fig. 4 ▸).

## Database survey   

A search of the Cambridge Structural Database (CSD Version 5.38; Groom *et al.*, 2016 ▸) for BODIPY (4,4-di­fluoro-4-bora-3a,4a-di­aza-*s*-indacenes) derivatives yielded 806 hits. Until 2001, there were only five reports [CSD refcode OCEBIL10 (Bonfiglio *et al.*, 1983 ▸), JEHFUX (Picou *et al.*, 1990 ▸), RETLUX (Kollmannsberger *et al.*, 1997 ▸), QAQTOR (Chen *et al.*, 1999 ▸) and XEJQAE (Burghart *et al.*, 1999 ▸)], but as the utility of BODIPY dyes was recognized, structural reports increased significantly. In all cases, the nearly planar BODIPY skeleton is modified with various functional groups, but no compound having a carb­oxy­lic acid directly attached to the BODIPY skeleton has been reported.

## Synthesis and crystallization   

The title compound was synthesized according to a previously described method (Giessler *et al.*, 2010 ▸; Bihovsky & Pendrak, 1996 ▸). The compound was purified by column chromatography. Single crystals were obtained by slow evaporation from a mixed solution of cyclo­hexa­ne/di­chloro­methane (1:1 *v* *v*) at room temperature.

## Refinement   

Crystal data, data collection and structure refinement details are summarized in Table 2 ▸. The H atom of the carboxyl group was refined freely, while the other H atoms were placed in geometrically idealized positions (C—H = 0.93–0.97 Å) and treated as riding on their parent atoms, with *U*_iso_(H) = 1.5*U*_eq_(C) for methyl H atoms and 1.2*U*_eq_(C) for methyl­ene and aromatic H atoms.

## Supplementary Material

Crystal structure: contains datablock(s) I. DOI: 10.1107/S2056989017016942/is5481sup1.cif

Structure factors: contains datablock(s) I. DOI: 10.1107/S2056989017016942/is5481Isup2.hkl

Supporting information file. DOI: 10.1107/S2056989017016942/is5481Isup3.cdx

CCDC reference: 1587383

Additional supporting information:  crystallographic information; 3D view; checkCIF report

## Figures and Tables

**Table 1:** Hydrogen-bond geometry (Å, °) *Cg*2 is the centroid of the N5/C5–C7/C10 ring.

*D*—H⋯*A*	*D*—H	H⋯*A*	*D*⋯*A*	*D*—H⋯*A*
C11—H11*A*⋯F1	0.96	2.52	3.146 (2)	123
C13—H13*B*⋯F1	0.97	2.49	3.096 (2)	120
C11—H11*B*⋯F2^i^	0.96	2.42	3.311 (2)	154
C6—H6⋯*Cg*2^i^	0.93	2.82	3.664 (1)	152
O16—H16⋯O15^ii^	0.91 (2)	1.75 (2)	2.648 (2)	175 (2)
C2—H2⋯O16^iii^	0.93	2.57	3.479 (2)	168
C14—H14*A*⋯F1^iv^	0.97	2.43	3.125 (2)	128

Symmetry codes: (i) 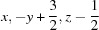; (ii) 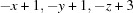; (iii) 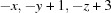; (iv) 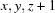.

**Table 2:** Experimental details

Crystal data	
Chemical formula	C_14_H_15_BF_2_N_2_O_2_
*M*_r_	292.09
Crystal system, space group	Monoclinic, *P*2_1_/*c*
Temperature (K)	100
*a*, *b*, *c* (Å)	7.9474 (3), 27.3202 (9), 6.3886 (2)
β (°)	103.903 (3)
*V* (Å^3^)	1346.48 (8)
*Z*	4
Radiation type	Cu *K*α
μ (mm^−1^)	0.97
Crystal size (mm)	0.35 × 0.17 × 0.13

Data collection	
Diffractometer	Rigaku Oxford Diffraction XtaLAB Pro: Kappa single and P200K
Absorption correction	Multi-scan (*CrysAlis PRO*; Rigaku Oxford Diffraction, 2015 ▸)
*T*_min_, *T*_max_	0.739, 0.878
No. of measured, independent and observed [*I* > 2σ(*I*)] reflections	7185, 2650, 2441
*R*_int_	0.024
(sin θ/λ)_max_ (Å^−1^)	0.624

Refinement	
*R*[*F* ^2^ > 2σ(*F* ^2^)], *wR*(*F* ^2^), *S*	0.035, 0.093, 1.06
No. of reflections	2650
No. of parameters	196
H-atom treatment	H atoms treated by a mixture of independent and constrained refinement
Δρ_max_, Δρ_min_ (e Å^−3^)	0.20, −0.19

Computer programs: *CrysAlis PRO* (Rigaku Oxford Diffraction, 2015 ▸), *SHELXS97* (Sheldrick, 2008 ▸), *SHELXL2016* (Sheldrick, 2015 ▸), *PLATON* (Spek, 2009 ▸) and *publCIF* (Westrip, 2010 ▸).

## supplementary crystallographic information

### Crystal data

**Table:**

C_14_H_15_BF_2_N_2_O_2_	*F*(000) = 608
*M**_r_* = 292.09	*D*_x_ = 1.441 Mg m^−^^3^
Monoclinic, *P*2_1_/*c*	Cu *K*α radiation, λ = 1.54184 Å
*a* = 7.9474 (3) Å	Cell parameters from 4875 reflections
*b* = 27.3202 (9) Å	θ = 6.6–73.9°
*c* = 6.3886 (2) Å	µ = 0.97 mm^−^^1^
β = 103.903 (3)°	*T* = 100 K
*V* = 1346.48 (8) Å^3^	Plate, red
*Z* = 4	0.35 × 0.17 × 0.13 mm

### Data collection

**Table:**

Rigaku Oxford Diffraction XtaLAB Pro: Kappa single and P200K diffractometer	2650 independent reflections
Radiation source: rotated anode	2441 reflections with *I* > 2σ(*I*)
Detector resolution: 8.336 pixels mm^-1^	*R*_int_ = 0.024
ω scans	θ_max_ = 74.1°, θ_min_ = 3.2°
Absorption correction: multi-scan (CrysAlis PRO; Rigaku Oxford Diffraction, 2015)	*h* = −9→9
*T*_min_ = 0.739, *T*_max_ = 0.878	*k* = −33→33
7185 measured reflections	*l* = −7→7

### Refinement

**Table:**

Refinement on *F*^2^	0 restraints
Least-squares matrix: full	Hydrogen site location: mixed
*R*[*F*^2^ > 2σ(*F*^2^)] = 0.035	H atoms treated by a mixture of independent and constrained refinement
*wR*(*F*^2^) = 0.093	*w* = 1/[σ^2^(*F*_o_^2^) + (0.0442*P*)^2^ + 0.5604*P*] where *P* = (*F*_o_^2^ + 2*F*_c_^2^)/3
*S* = 1.06	(Δ/σ)_max_ = 0.001
2650 reflections	Δρ_max_ = 0.20 e Å^−^^3^
196 parameters	Δρ_min_ = −0.19 e Å^−^^3^

### Special details

**Table:**

Geometry. All esds (except the esd in the dihedral angle between two l.s. planes) are estimated using the full covariance matrix. The cell esds are taken into account individually in the estimation of esds in distances, angles and torsion angles; correlations between esds in cell parameters are only used when they are defined by crystal symmetry. An approximate (isotropic) treatment of cell esds is used for estimating esds involving l.s. planes.

### Fractional atomic coordinates and isotropic or equivalent isotropic displacement parameters (Å^2^)

**Table:**

	*x*	*y*	*z*	*U*_iso_*/*U*_eq_	
F1	0.13158 (9)	0.60985 (3)	0.65315 (12)	0.0255 (2)	
F2	0.08937 (9)	0.67726 (3)	0.84017 (12)	0.02436 (19)	
B4	0.00668 (17)	0.64191 (5)	0.6943 (2)	0.0198 (3)	
N4	−0.12985 (13)	0.61414 (4)	0.78542 (17)	0.0199 (2)	
N5	−0.09242 (13)	0.66655 (4)	0.48014 (16)	0.0191 (2)	
O15	0.39633 (12)	0.54006 (4)	1.32206 (16)	0.0290 (2)	
O16	0.28087 (13)	0.49056 (4)	1.52940 (16)	0.0290 (2)	
H16	0.395 (3)	0.4817 (8)	1.583 (4)	0.060 (6)*	
C1	−0.38942 (17)	0.58795 (5)	0.8312 (2)	0.0247 (3)	
H1	−0.507975	0.582922	0.811031	0.030*	
C2	−0.26001 (17)	0.56942 (5)	0.9971 (2)	0.0251 (3)	
H2	−0.275726	0.549606	1.109267	0.030*	
C3	−0.10063 (17)	0.58593 (5)	0.9655 (2)	0.0218 (3)	
C5	−0.02424 (16)	0.69272 (5)	0.3422 (2)	0.0207 (3)	
C6	−0.15801 (17)	0.70826 (5)	0.1669 (2)	0.0224 (3)	
H6	−0.143874	0.726714	0.049951	0.027*	
C7	−0.31272 (17)	0.69140 (5)	0.1990 (2)	0.0221 (3)	
C8	−0.37630 (16)	0.64040 (5)	0.5064 (2)	0.0217 (3)	
H8	−0.495594	0.640225	0.449840	0.026*	
C9	−0.30802 (16)	0.61578 (5)	0.6990 (2)	0.0212 (3)	
C10	−0.27228 (16)	0.66503 (5)	0.3973 (2)	0.0204 (3)	
C11	0.16412 (16)	0.70348 (5)	0.3788 (2)	0.0240 (3)	
H11A	0.225357	0.688706	0.511699	0.036*	
H11B	0.181863	0.738252	0.386612	0.036*	
H11C	0.206667	0.690412	0.261876	0.036*	
C12	−0.48969 (17)	0.69906 (6)	0.0566 (2)	0.0284 (3)	
H12A	−0.547730	0.724511	0.115597	0.043*	
H12B	−0.554999	0.669256	0.047816	0.043*	
H12C	−0.479989	0.708339	−0.084927	0.043*	
C13	0.07910 (17)	0.57591 (5)	1.0954 (2)	0.0251 (3)	
H13A	0.128247	0.606001	1.164537	0.030*	
H13B	0.149880	0.565559	0.999090	0.030*	
C14	0.08684 (17)	0.53691 (5)	1.2669 (2)	0.0230 (3)	
H14A	0.029454	0.548953	1.374614	0.028*	
H14B	0.025020	0.508035	1.201109	0.028*	
C15	0.26966 (17)	0.52318 (5)	1.3743 (2)	0.0235 (3)	

### Atomic displacement parameters (Å^2^)

**Table:**

	*U*^11^	*U*^22^	*U*^33^	*U*^12^	*U*^13^	*U*^23^
F1	0.0230 (4)	0.0268 (4)	0.0273 (4)	0.0073 (3)	0.0073 (3)	0.0027 (3)
F2	0.0249 (4)	0.0249 (4)	0.0224 (4)	−0.0054 (3)	0.0041 (3)	−0.0017 (3)
B4	0.0182 (6)	0.0208 (7)	0.0202 (7)	0.0013 (5)	0.0043 (5)	0.0005 (5)
N4	0.0197 (5)	0.0185 (5)	0.0210 (5)	0.0004 (4)	0.0040 (4)	0.0006 (4)
N5	0.0181 (5)	0.0197 (5)	0.0201 (5)	0.0009 (4)	0.0057 (4)	0.0000 (4)
O15	0.0257 (5)	0.0280 (5)	0.0309 (5)	−0.0027 (4)	0.0021 (4)	0.0099 (4)
O16	0.0272 (5)	0.0309 (5)	0.0283 (5)	0.0031 (4)	0.0058 (4)	0.0120 (4)
C1	0.0222 (6)	0.0252 (7)	0.0279 (7)	−0.0020 (5)	0.0081 (5)	0.0016 (5)
C2	0.0281 (7)	0.0235 (6)	0.0246 (7)	−0.0020 (5)	0.0079 (5)	0.0036 (5)
C3	0.0257 (6)	0.0181 (6)	0.0214 (6)	−0.0003 (5)	0.0054 (5)	0.0002 (5)
C5	0.0219 (6)	0.0194 (6)	0.0222 (6)	0.0016 (5)	0.0078 (5)	−0.0005 (5)
C6	0.0245 (6)	0.0228 (6)	0.0210 (6)	0.0029 (5)	0.0077 (5)	0.0031 (5)
C7	0.0225 (6)	0.0223 (6)	0.0213 (6)	0.0035 (5)	0.0053 (5)	−0.0005 (5)
C8	0.0179 (6)	0.0224 (6)	0.0244 (6)	0.0009 (5)	0.0045 (5)	−0.0012 (5)
C9	0.0187 (6)	0.0212 (6)	0.0239 (6)	0.0001 (5)	0.0054 (5)	−0.0005 (5)
C10	0.0182 (6)	0.0215 (6)	0.0215 (6)	0.0025 (5)	0.0045 (5)	−0.0006 (5)
C11	0.0208 (6)	0.0251 (7)	0.0274 (7)	−0.0006 (5)	0.0081 (5)	0.0014 (5)
C12	0.0224 (6)	0.0363 (8)	0.0258 (7)	0.0057 (6)	0.0044 (5)	0.0046 (6)
C13	0.0246 (6)	0.0223 (6)	0.0262 (7)	−0.0015 (5)	0.0016 (5)	0.0041 (5)
C14	0.0258 (6)	0.0207 (6)	0.0215 (6)	−0.0002 (5)	0.0035 (5)	0.0000 (5)
C15	0.0290 (7)	0.0193 (6)	0.0209 (6)	−0.0012 (5)	0.0034 (5)	0.0008 (5)

### Geometric parameters (Å, º)

**Table:**

F1—B4	1.3953 (15)	C6—C7	1.3738 (18)
F2—B4	1.3926 (16)	C6—H6	0.9300
B4—N4	1.5479 (17)	C7—C10	1.4253 (18)
B4—N5	1.5579 (17)	C7—C12	1.4948 (18)
N4—C3	1.3575 (16)	C8—C10	1.3781 (18)
N4—C9	1.3915 (16)	C8—C9	1.3926 (18)
N5—C5	1.3458 (16)	C8—H8	0.9300
N5—C10	1.4006 (16)	C11—H11A	0.9600
O15—C15	1.2243 (16)	C11—H11B	0.9600
O16—C15	1.3197 (16)	C11—H11C	0.9600
O16—H16	0.92 (2)	C12—H12A	0.9600
C1—C2	1.3833 (19)	C12—H12B	0.9600
C1—C9	1.4047 (18)	C12—H12C	0.9600
C1—H1	0.9300	C13—C14	1.5189 (18)
C2—C3	1.4042 (18)	C13—H13A	0.9700
C2—H2	0.9300	C13—H13B	0.9700
C3—C13	1.4948 (18)	C14—C15	1.4978 (18)
C5—C6	1.4117 (18)	C14—H14A	0.9700
C5—C11	1.4878 (17)	C14—H14B	0.9700
			
F2—B4—F1	108.59 (10)	N4—C9—C8	120.64 (11)
F2—B4—N4	110.42 (10)	N4—C9—C1	108.39 (11)
F1—B4—N4	110.96 (10)	C8—C9—C1	130.90 (12)
F2—B4—N5	110.23 (10)	C8—C10—N5	120.33 (11)
F1—B4—N5	109.78 (10)	C8—C10—C7	131.45 (12)
N4—B4—N5	106.86 (10)	N5—C10—C7	108.22 (11)
C3—N4—C9	107.77 (11)	C5—C11—H11A	109.5
C3—N4—B4	127.19 (11)	C5—C11—H11B	109.5
C9—N4—B4	124.98 (10)	H11A—C11—H11B	109.5
C5—N5—C10	107.65 (10)	C5—C11—H11C	109.5
C5—N5—B4	127.34 (10)	H11A—C11—H11C	109.5
C10—N5—B4	125.02 (10)	H11B—C11—H11C	109.5
C15—O16—H16	109.7 (14)	C7—C12—H12A	109.5
C2—C1—C9	107.09 (12)	C7—C12—H12B	109.5
C2—C1—H1	126.5	H12A—C12—H12B	109.5
C9—C1—H1	126.5	C7—C12—H12C	109.5
C1—C2—C3	107.61 (11)	H12A—C12—H12C	109.5
C1—C2—H2	126.2	H12B—C12—H12C	109.5
C3—C2—H2	126.2	C3—C13—C14	113.36 (11)
N4—C3—C2	109.13 (11)	C3—C13—H13A	108.9
N4—C3—C13	121.36 (11)	C14—C13—H13A	108.9
C2—C3—C13	129.50 (12)	C3—C13—H13B	108.9
N5—C5—C6	109.54 (11)	C14—C13—H13B	108.9
N5—C5—C11	123.37 (12)	H13A—C13—H13B	107.7
C6—C5—C11	127.08 (12)	C15—C14—C13	111.89 (11)
C7—C6—C5	108.12 (11)	C15—C14—H14A	109.2
C7—C6—H6	125.9	C13—C14—H14A	109.2
C5—C6—H6	125.9	C15—C14—H14B	109.2
C6—C7—C10	106.47 (11)	C13—C14—H14B	109.2
C6—C7—C12	127.39 (12)	H14A—C14—H14B	107.9
C10—C7—C12	126.14 (12)	O15—C15—O16	123.19 (12)
C10—C8—C9	121.93 (12)	O15—C15—C14	123.47 (12)
C10—C8—H8	119.0	O16—C15—C14	113.34 (11)
C9—C8—H8	119.0		
			
F2—B4—N4—C3	−62.77 (16)	C5—C6—C7—C10	−0.11 (15)
F1—B4—N4—C3	57.69 (16)	C5—C6—C7—C12	179.56 (13)
N5—B4—N4—C3	177.34 (11)	C3—N4—C9—C8	−176.99 (11)
F2—B4—N4—C9	114.21 (13)	B4—N4—C9—C8	5.53 (19)
F1—B4—N4—C9	−125.33 (12)	C3—N4—C9—C1	0.32 (14)
N5—B4—N4—C9	−5.68 (16)	B4—N4—C9—C1	−177.16 (11)
F2—B4—N5—C5	62.94 (16)	C10—C8—C9—N4	−1.75 (19)
F1—B4—N5—C5	−56.64 (16)	C10—C8—C9—C1	−178.37 (13)
N4—B4—N5—C5	−177.05 (11)	C2—C1—C9—N4	−0.17 (15)
F2—B4—N5—C10	−117.07 (12)	C2—C1—C9—C8	176.77 (13)
F1—B4—N5—C10	123.35 (12)	C9—C8—C10—N5	−0.94 (19)
N4—B4—N5—C10	2.94 (16)	C9—C8—C10—C7	178.50 (13)
C9—C1—C2—C3	−0.03 (15)	C5—N5—C10—C8	179.99 (11)
C9—N4—C3—C2	−0.34 (14)	B4—N5—C10—C8	0.00 (18)
B4—N4—C3—C2	177.06 (12)	C5—N5—C10—C7	0.43 (14)
C9—N4—C3—C13	178.81 (11)	B4—N5—C10—C7	−179.55 (11)
B4—N4—C3—C13	−3.79 (19)	C6—C7—C10—C8	−179.68 (13)
C1—C2—C3—N4	0.23 (15)	C12—C7—C10—C8	0.6 (2)
C1—C2—C3—C13	−178.83 (13)	C6—C7—C10—N5	−0.19 (14)
C10—N5—C5—C6	−0.51 (14)	C12—C7—C10—N5	−179.87 (12)
B4—N5—C5—C6	179.48 (11)	N4—C3—C13—C14	−170.06 (11)
C10—N5—C5—C11	178.33 (11)	C2—C3—C13—C14	8.9 (2)
B4—N5—C5—C11	−1.69 (19)	C3—C13—C14—C15	172.63 (11)
N5—C5—C6—C7	0.39 (15)	C13—C14—C15—O15	−3.02 (18)
C11—C5—C6—C7	−178.39 (12)	C13—C14—C15—O16	177.79 (11)

### Hydrogen-bond geometry (Å, º)

*Cg*2 is the centroid of the N5/C5–C7/C10 ring.

**Table:**

*D*—H···*A*	*D*—H	H···*A*	*D*···*A*	*D*—H···*A*
C11—H11*A*···F1	0.96	2.52	3.146 (2)	123
C13—H13*B*···F1	0.97	2.49	3.096 (2)	120
C11—H11*B*···F2^i^	0.96	2.42	3.311 (2)	154
C6—H6···*Cg*2^i^	0.93	2.82	3.664 (1)	152
O16—H16···O15^ii^	0.91 (2)	1.75 (2)	2.648 (2)	175 (2)
C2—H2···O16^iii^	0.93	2.57	3.479 (2)	168
C14—H14*A*···F1^iv^	0.97	2.43	3.125 (2)	128

Symmetry codes: (i) *x*, −*y*+3/2, *z*−1/2; (ii) −*x*+1, −*y*+1, −*z*+3; (iii) −*x*, −*y*+1, −*z*+3; (iv) *x*, *y*, *z*+1.
